# Development of an Inverted Epifluorescence Microscope for Long-Term Monitoring of Bacteria in Multiplexed Microfluidic Devices

**DOI:** 10.3390/s20154140

**Published:** 2020-07-25

**Authors:** Amaro Torres-Simón, María Henar Marino, Clara Gómez-Cruz, Marina Cañadas, Miguel Marco, Jorge Ripoll, Juan José Vaquero, Arrate Muñoz-Barrutia

**Affiliations:** 1 Departamento de Bioingeniería e Ingeniería Aeroespacial, Universidad Carlos III de Madrid, 28911 Leganés, Spain; amaro.torres@uc3m.es (A.T.-S.); 100363389@alumnos.uc3m.es (M.H.M.); clgomezc@pa.uc3m.es (C.G.-C.); macanada@pa.uc3m.es (M.C.); jorge.ripoll@uc3m.es (J.R.); juanjose.vaquero@uc3m.es (J.J.V.); 2 Departamento de Ingeniería Mecánica, Universidad Carlos III de Madrid, 28911 Leganés, Spain; miguel.marco@uc3m.es; 3 Instituto de Investigación Sanitaria Gregorio Marañón, 28009 Madrid, Spain

**Keywords:** epifluorescence microscopy, time-lapse microscopy, fluorescence imaging, single-cell analysis, microfluidics, GFP-expressing bacteria, drug development, antibiotic resistance

## Abstract

Developing more efficient methods for antibiotic susceptibility testing is a pressing issue in novel drug development as bacterial resistance to antibiotics becomes increasingly common. Microfluidic devices have been demonstrated to be powerful platforms that allow researchers to perform multiplexed antibiotic testing. However, the level of multiplexing within microdevices is limited, evidencing the need of creating simple, low-cost and high-resolution imaging systems that can be integrated in antibiotic development pipelines. This paper describes the design and development of an epifluorescence inverted microscope that enables long-term monitoring of bacteria inside multiplexed microfluidic devices. The goal of this work is to provide a simple microscope powerful enough to allow single-cell analysis of bacteria at a reduced cost. This facilitates increasing the number of microscopes that are simultaneously used for antibiotic testing. We prove that the designed system is able to accurately detect fluorescent beads of 100 nm, demonstrating comparable features to high-end commercial microscopes and effectively achieving the resolution required for single-cell analysis of bacteria. The proposed microscope could thus increase the efficiency in antibiotic testing while reducing cost, size, weight, and power requirements, contributing to the successful development of new antibiotic drugs.

## 1. Introduction

Multidrug resistant bacterial diseases have become a major concern worldwide. Its causes are primarily found in treatment mismanagement, direct transmission of the resistant pathogens, and rapid spontaneous mutation [1]. One of the main problems associated with multidrug bacterial resistance is the fact that, currently, there is not a fast enough diagnostic procedure to guide the prescription of the optimal antibiotic for a specific disease. The standard procedures carried out require taking the patient’s samples to a microbiology lab and waiting several days to obtain a pure culture of the pathogen [2]. A faster approach could involve the use of lab-on-a-chip technology, which could guide antibiotic prescriptions in a much efficient way [3]. Besides solutions at the patient level, new drugs or regimes combining several drugs are essential to tackle new strains of drug-resistant bacteria; however, in vitro testing all possible candidates is excessively time-consuming and resource-intensive. To address this issue, microfluidic devices are being used to evaluate several drugs concurrently, speeding in this way the testing without raising the resources employed [4,5].

The aforementioned tests are based on the growth analysis of bacteria modified to overexpress different fluorescent proteins, among which the most used is Green Fluorescence Protein (GFP) [6,7,8]. As only living bacteria are actively producing fluorescent proteins, the underlying principle for the use of these bacteria is the correlation between the intensity of the emitted fluorescence and the number of living bacteria present in a sample [9]. For this reason, a fluorescence microscope can be used to quickly quantify bacterial population dynamics [10]. 

Time-lapse fluorescence microscopy is a widely used method in the first stages of antibiotic testing due to the simplicity of the quantification required. Antibiotic efficiency can thus be numerically assessed by the proportion of bacteria that remain alive in the presence of the drug [11]. Typical antibiotic testing implies culturing bacteria in the presence of the antibiotic for weeks. Then, the number of remaining bacteria is quantified by culturing them elsewhere and waiting to count the number of bacterial colonies that have appeared. Each colony is expected to have come from one colony-forming unit (CFU), a standard way of measuring the number of viable bacteria [12]. The use of fluorescence microscopy eliminates the need for the second culture period by quantifying the number of bacteria in real-time. 

Prior to the chain of trials that will develop a drug that is potent and safe enough for the clinical application, thousands of potential compounds need to be tested in order to choose the optimal candidate [13]. One of the solutions that is being implemented by research groups to accelerate this selection process consists on the development of multiplexed microfluidic devices for antibiotic testing [14]. Relevant examples can be found in the articles published by Manina et al. [15], He et al. [16], Mohan et al. [17], and Wistrand-Yuen et al. [18]. However, multiplexing on the microfluidic device is conditioned by the dimensions of the microscope stage opening as it determines the area that can be swept during acquisition. A possible solution to this problem is to run experiments in parallel if several microscopes are available [19]. Namely, multiplexing the microscope acquisition besides multiplexing at the microfluidic device level. The main limitation of this approach is the high cost of commercial fluorescence microscopes. 

An alternative solution for multiplexing the imaging capacity is the development of custom made special-purpose microscopes [20]. In the case of a small-scale production of microscopes for specific research purposes, a great cost reduction can be achieved by assembling commercially available parts. Besides, this mode of production also ensures that only the combination of features required is implemented in the microscope. 

Among the components required for this application are high magnification objectives, with good numerical apertures in order to resolve details in the range from hundreds of nanometers to few microns. This level of resolution warrants the correct visualization of bacteria required for drug screening inside microfluidic devices. The microscope also needs to be able to perform time-lapse microscopy during long drug assays in order to follow the number of living bacteria in the presence of the antibiotic for periods of time of up to two weeks. 

To accelerate these susceptibility testing of antibiotics we developed a robust, moderate cost, high-resolution imaging system. The aim of this microscope is to serve as an imaging platform that enables single-cell visualization, and the collection and analysis of large sets of fluorescence microscopy images. Specifically, the epifluorescence microscope has been designed and optimized for imaging GFP expressing bacteria cultured inside polydimethylsiloxane (PDMS) microfluidic chips. The imaging system allows automated data collection in time-lapse mode over long periods of time. Time-lapse videos are a powerful tool to monitor and assess the susceptibility of bacterial cultures; particularly in the presence of novel antibiotics. An important advantage over standard commercially available microscopes is the fact that the available imaging area is considerably larger, which means that a higher degree of multiplexing at the microdevice level can be achieved. 

The proposed imaging system is an epifluorescence microscope with an inverted configuration. It comprises a light-sensitive sensor that allows image acquisition of the bacteria inside microfluidic devices for antibiotic susceptibility tests. The epifluorescence configuration ensures that the light passes through the sample at a straight angle, allowing the light beam to deeply penetrate the sample and maximizing illumination. This provides intense signals, enabling real-time visualization of sub-cellular processes that provide essential information about antibiotic mechanisms of action and facilitating co-localization studies in 2D [21,22]. The inverted configuration of the microscope minimizes the distance between the samples, that sit on top of the coverslip that seals the microfluidic device, and the objective. This is crucial for imaging bacteria inside microdevices, in which the distance between the top of the microdevice and the microfluidic channels is higher than the working distance of the majority of the high-magnification objectives.

Finally, the sensor employed for image acquisition plays a central role, as specific characteristics are essential for the detection and analysis of the GFP-emitting bacterial colonies during susceptibility tests. A high-quality sensor coupled to a consistent set of lenses and objectives is crucial for the imaging system. The ideal sensor for this application enumerates several requisites, being the most critical one a high quantum efficiency and a high signal-to-noise ratio to provide excellent sensitivity, essential for detection of low-light fluorescence or minor changes in fluorescence. Such sensitivity is required to capture as much information as possible while reducing excitation intensity and exposure time to avoid phototoxicity and photobleaching when imaging living organisms. Additionally, a monochrome camera is a more suitable sensor for fluorescence imaging than a color one. When a single wavelength is being imaged at the same time, the lack of color filter allows a higher number of photons to arrive to the sensor, thus increasing its resolution. 

## 2. Materials and Methods

### 2.1. Imaging System Design and Components

**Figure 1:**
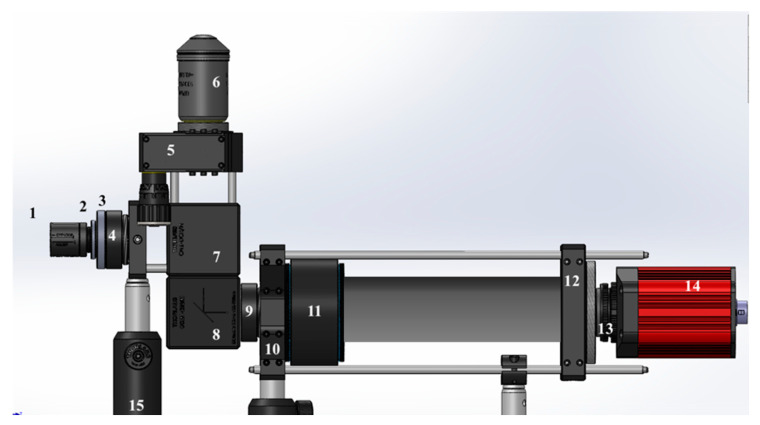
Schematic view of the imaging system components. CAD files for the components were obtained from the Thorlabs webpage and reproduced with permission. Copyright © 2020 Thorlabs. CAD files were assembled using SolidWorks. The numbered imaging components are listed in Table 1.

The custom-made epifluorescence inverted microscope configuration is presented in Figure 1 and the individual components are listed in Table 1. 

#### 2.1.1. General Setup

As previously explained, the imaging system was designed for antibiotic susceptibility testing. Experiments with potentially harmful bacteria must be performed inside biosecurity laboratories (BSL-3 or BSL-4), where space is limited. Consequently, one of the main concerns was to ensure a compact design, in order to maximize space availability and allow the integration of multiple imaging systems in the same laboratory. The microscope was mounted on an optical table (Thorlabs MB4545/M) of dimensions 45 × 45 cm. This table allowed adjusting distances between components and reduced external and internal vibrations to maximize image quality and avoid movement artifacts. Additionally, the system comprises an XY stage (Prior ProScan II) located above the objective (corresponding to an inverted configuration), and a sample holder (Prior ProScan II sample holder) coupled to a custom-made 3D printed piece to grip the manufactured microfluidic chips in place and mount them into the XY stage. The components of the imaging system were mounted and attached to the optical table by the Thorlabs cage system and post holders. The total cost of the imaging system was kept under 15k €, ensuring a notable reduction compared to commercial systems with similar features. 

#### 2.1.2. Excitation Path

Light coming from a blue LED (LUMILEDS blue light-emitting diode) enters the microscope and is homogenized by a glass ground diffuser (Thorlabs Unmounted N-BK7 Ground Glass Diffuser DG10-220). The diverging beam is condensed by a 30 mm focal length achromatic doublets lens (Thorlabs AC254-030-A). To control the intensity and frequency of the LED illumination a custom-made electronic circuit was designed.

In microfluidics research, the fluorescence signal mainly comes from cells either tagged with a fluorescent antibody or modified to express a particular fluorochrome, generally enhanced green fluorescent protein (eGFP). To simplify the design and reduce hardware costs, the microscope was built to detect light emitted in the green spectrum (502–550 nm), as the majority of fluorochromes used emit in this range. To ensure that only light in the required exciting wavelengths reached the sample, an emission filter (Thorlabs MF469-35) was placed in the beam path. This filter allowed light with a center wavelength (CWL) of 469 nm and a bandwidth (FWHM) of 35 nm. This pattern of excitation matched the requirements for eGFP and other green-emitting fluorophores. The filtered light beam is reflected by a dichroic mirror (Thorlabs MD498) to enter the electrically tunable lens (Optotune EL-16-40-TC) and magnification objective in a straight angle. The microscope includes two possible magnification objectives, which can be manually exchanged depending on the application requirements. The first one is the Motic CCIS Plan achromatic phase objective UC Ph2 20× objective, with numerical aperture of 0.45 and working distance of 0.8 mm; this objective is used to capture entire sections of microchannels and to facilitate focusing the sample. The second objective is the Olympus UPLFLN 100× (Oil immersion), which provides a numerical aperture of 1.3 and a working distance of 0.2 mm. This high magnification combined with the high numerical aperture is a valuable addition to the imaging system as it allows the visualization of details at the cellular and subcellular level with high resolution.

#### 2.1.3. Emission Path

The epifluorescence set up enables part of the light emitted by the illuminated sample to be collected by the same magnification objective [23]. The collimated beam travels backwards through the same optical path, passing through the dichroic mirror. A dielectric turning mirror (Thorlabs CCM1-E02/M 30) reflects the beam to focus it on the detector. The emitted light passes through a band pass emission filter (Thorlabs MF525-35) to ensure that only fluorescence signals in the green spectrum (502–550 nm) coming from the excited sample reach the detector, eliminating any residual excitation light. After filtering, the beam passes through a tube lens (Thorlabs TTL180-A) that focuses the parallel beams, that exit from the objective, into the digital sensor.

The sensor used in the imaging system is a Manta G-145B NIR, a near infrared optimized GigE camera with a Sony ICX285 Charged Coupled Devices (CCD) sensor. In these Charged Coupled Devices, some of the energy from striking photons emitted by the sample when irradiated with the exciting laser is absorbed within a semiconductor material. The energy absorbed liberates electrons creating an electric current. In CCD, unlike in CMOS (Complementary Metal Oxide Semiconductor) sensors, the electric current generated is converted to a voltage at an output node, instead of inside each pixel. The output is a voltage proportional to the intensity of the incoming light. A CCD (Charge Coupled Devices) camera was selected for this work, as it yields a good quality-price ratio for our specific application. Antibiotic susceptibility tests rely on the quantification of fluorescence intensity to assess the effect of certain drug on the bacterial colony. To that end, the selected CCD sensor has a high sensitivity that allows the detection of low-light levels while providing a high enough resolution to capture bacterial morphology, relevant for scientists and researchers to study the mechanism of action of the tested drug. The flexibility of the imaging system configuration allows the implementation of digital sensors that fulfilled the requirements of different drug development applications.

**Figure 2:**
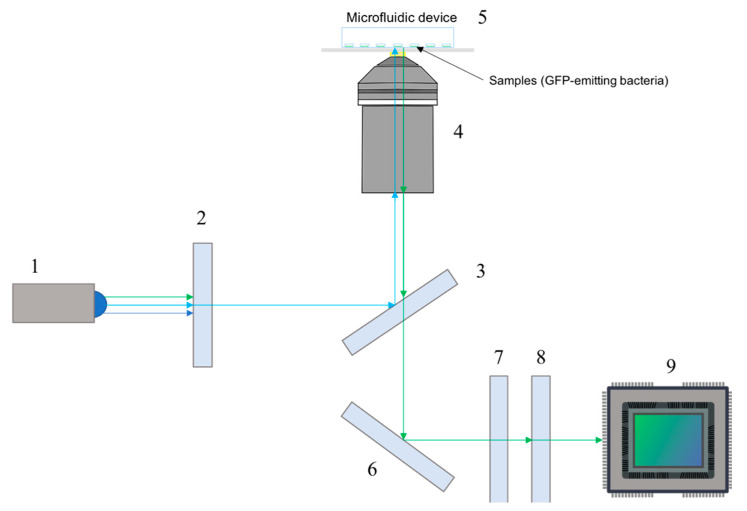
Schematic of the excitation and emission light paths. Components depicted are: (1) LED, (2) excitation filter, (3) dichroic mirror, (4) objective, (5) microfluidic device, (6) dielectric mirror, (7) emission filter, (8) tube lens, (9) fluorescence digital sensor.

A schematic of the emission and excitation paths is depicted in Figure 2.

#### 2.1.4. Software

The software used for image acquisition was the Manta G-145B camera manufacturer’s software, VIMBA viewer (Vimba Viewer API version 1.7.0). Images were processed with ImageJ (ImageJ bundled with 64-bit Java 1.8.0) [24]. ImageJ was also used to merge obtained bright-field and fluorescence images from the time-lapse microscopy experiments.

### 2.2. Microdevice Fabrication

Microfluidic chips were fabricated using standard soft lithography techniques. Briefly, the designed microchannels made with AutoCAD were transferred to a SU-8 sheet spin-coated to the desired height by applying UV light through a negative patterned mask. The wafer was used to cast PDMS using soft lithography. PDMS (Dow and Corning’s Sylgard 184) in a 10:1 proportion between elastomer and curing agent is poured on the wafer, baked (1 h at 70 °C) and demolded. Following, inlet and outlet holes were performed with a puncher (diameter: 2 mm) to later introduce and seal with PDMS the silicon tubes (diameter: 0.76 mm) that transport the fluid in and out the microfluidic chip. After curing and cleaning the surfaces, PDMS chips were sealed to a glass coverslip using air plasma treatment. Both glass and PDMS are optically transparent materials which ensure that the microdevices can be used for microscopy. The low autofluorescence of PDMS makes it ideal for the fabrication of microdevices intended for fluorescence microscopy [25]. The microdevices were manufactured using standard microscopy coverslips to ensure that the samples were always within the working distance of the objective. 

### 2.3. Time-Lapse Imaging of Fluorescent Beads

Time-lapse images of microdevices were obtained using the proposed microscope setup. A solution of 1:1000 of 1 μm diameter polymer microspheres with green fluorescence (Duke Scientific Corp.) in distilled colored water was loaded into zero-flow microfluidic devices at a rate of 5 µl/min using a peristaltic pump (ISMATEC IPC ISM932) until all the chambers were filled. Colored water was used for these experiments in order to increase the contrast between empty and filled channels in bright-field images. 

To focus the samples, the stage was manually displaced in the axial direction to match the objective being used and then samples were fine focused by modulating the current passing through the electrically tunable lens (Optotune EL-16-40-TC) using the Optotune software. Navigation through the sample in the lateral directions to image the different channels was performed using the XY stage (Prior ProScan II) manual controller. 

For time-lapse imaging, a bright-field and a fluorescent image were acquired every 5 min using the camera software. In the case of the bright-field images, ambient illumination was used, with an exposure time of 40,000 µs and a gain of 17 db. For fluorescent images, the blue LED was used at its full power in the case of experiments with beads and a exposure time of 450,000 µs and a gain of 23 db. For imaging living cells and due to phototoxicity concerns, an Arduino program was developed to control the laser power, in order to ensure that the laser was only functioning during the time needed and at the minimum power required to correctly image the cells. 

### 2.4. Cell Culture for Single-Cell Analysis Applications

Human fluorescent fibroblasts were transfected with the vector pLZRS-IRES-EGFP, a mutation that makes the cells express cytoplasmatic green fluorescence [26]. Then, the cells were cultured on top of a glass slide for four days at 37 °C and 5% CO_2_ in a humidified chamber prior to imaging. DMEM medium (Invitrogen Life Technologies) supplemented with 10% fetal bovine serum (Thermo Scientific HyClone) and 1% antibiotic/antimycotic (Thermo Scientific Hyclone) was used as culture media, changed every two days. Once the appropriate confluence was reached, cells were fixed adding ice-cold methanol for 10 min, then washed with phosphate buffered saline (Thermo Fisher Scientific) prior to placing the coverslip onto a microscope slide using mounting solution.

## 3. Results

### 3.1. Real Setup and Microscope Characterization

**Figure 3:**
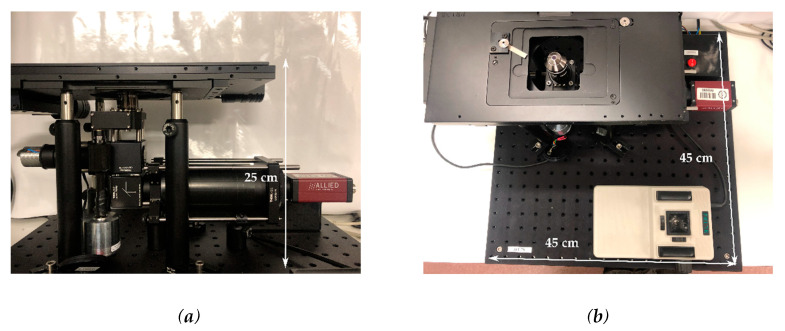
Final set up of the microscope; (**a**) lateral view and (**b**) top view.

The final configuration of the microscope is shown in Figure 3. The device final dimensions were 25 × 45 × 45 cm, in line with the objective of minimizing bulk space taken in BSL-3 laboratories. The dimensions of the imaging aperture were 67 × 73 mm which could accommodate two microscope slides side by side, doubling the standard imaging area for commercial microscopes. With this aperture, up to six microfluidic PDMS chips with dimensions 24 × 24 mm could be imaged concurrently. 

Although the field of view of the microscope was 445.05 × 334.76 µm using the 20× magnification objective, this bigger imaging aperture combined with the lateral movement of the XY stage (Prior ProScan II) allowed for point visiting of multiple channels during the same time-lapse experiment, effectively doubling the degree of multiplexing achievable in one microscope by doubling the imaging area. 

**Figure 4:**
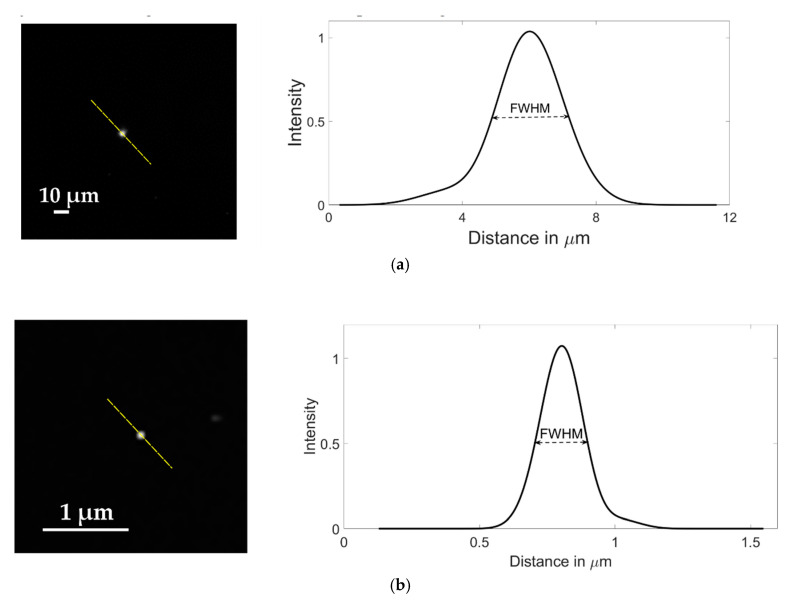
Images of fluorescent microspheres used to estimate the spatial resolution of the imaging system; (**a**) 1.0 µm diameter microsphere imaged with a Motic CCIS Plan achromatic phase objective UC Ph2 20× objective; (**b**) 100 nm diameter microsphere imaged with an Olympus UPLFLN 100× magnification objective. The graphs represent the Gaussian function that fits the average microsphere profile, taken along the diagonals passing through the highest intensity pixel (dashed lines).

The resolution of the instrument was characterized using the Point Spread Function (PSF) of fluorescent microspheres of 1 μm and 100 nm diameter. Spatial resolution of an optical system can be determined by measuring the full width at half maximum (FWHM) of the PSF of a fluorescent microsphere [27]. The microspheres were diluted to 1:1000 from the stock concentration in distilled water, with no other modifications and then imaged using the LUMILEDS blue light-emitting diode. To ensure that the resolution obtained was significant for later experiments, the beads were imaged in suspension inside a microfluidic device with channels of 35 μm in height to replicate the real conditions in which further imaging will be obtained. Typical images of microspheres samples are shown in Figure 4. From fluorescent cross sections of imaged microspheres, the obtained FWHM, and thus, the spatial resolution of our system was 2.34 μm for the images with 20× magnification using 1 μm diameter microspheres (Figure 4a) and 203.6 nm for the ones obtained with 100× magnification using 100 nm diameter microspheres (Figure 4b). 

### 3.2. Time-Lapse Imaging of The Microfluidic Devices

**Figure 5:**
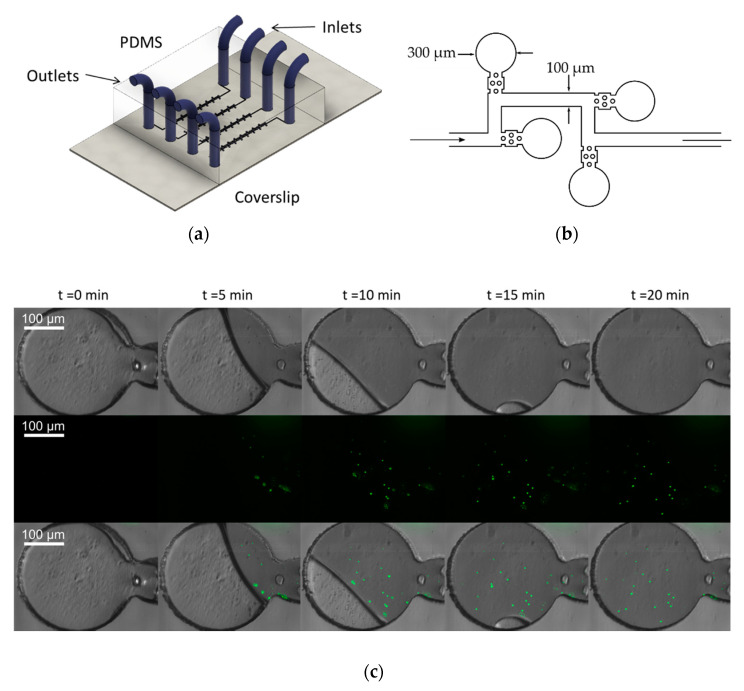
Time-lapse images of 1 μm fluorescence beads entering microchambers using a Motic CCIS Plan achromatic phase contrast objective UC Ph2 20× objective; (**a**) schematics of the microfluidic device for time-lapse microscopy of fluorescent bacteria; (**b**) detail of the microfluidic channel design (adapted from [28]). The chambers are 300 µm in diameter and the channels are 100 µm wide. The height of all the channels is 35 µm; (**c**) time-lapse images of 1 µm microspheres loaded into the microdevice. (Top Row) Bright-field channel; (Second Row) fluorescence channel and (Bottom Row) an ImageJ composite of bright-field and fluorescence channels are shown.

Time-lapse microscopy is among the most used methods to study bacterial population dynamics. To test the capabilities of the microscope for this kind of assays, we performed an experiment in which zero-flow chambers were filled with a suspension of 1 μm fluorescence microspheres in colored water (microdevice design adapted from [28]). Images of relevant time stamps (Figure 5) depict how the microbeads solution filled the zero-flow microchambers. It took 20 min to fill completely the chambers. Using these results from fluorescent microbeads, we expect that the imaging system to allow long-term monitoring of bacterial populations subjected to a drug or drug cocktails, as PDMS has been previously shown to be biocompatible in the long term [29] and the microscope could be used for prolonged periods of time without leading to phototoxicity or bleaching due to the Arduino control of the laser power. If successful, time-lapse videos obtained during antibiotic testing would be a powerful tool for researchers to assess the effectiveness of the drug in vitro. 

### 3.3. Applications for Single-Cell Analysis

Once the microscope was characterized and the ability to perform time-lapse experiments on microfluidic devices proven, it was necessary to demonstrate the ability of the developed system to visualize and thus analyze the morphology of the microorganisms of interest. This morphological analysis is an essential requirement for the study of antibiotic resistance carried out during drug development, as it allows to retrieve very valuable information about pharmacological effectiveness and the mechanism of action underlying bacterial death.

The proposed imaging system was tested in fixed cells and was able to differentiate intracellular components. Although the main objective was the imaging of bacterial cells, this result seems promising for using the microscope for cellular applications, potentially providing meaningful information about the morphology, allowing to study both the physiological and the disease conditions.

**Figure 6:**
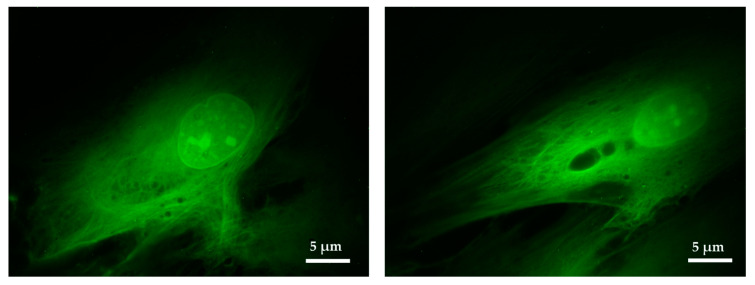
Close up visualization of fibroblast cells. Details of the fibers composing the cellular cytoskeleton and other intracellular structures can be appreciated. Images obtained with the Olympus UPLFLN 100× and processed using ImageJ.

To analyze the effect of antibiotics in a bacterial population, it is necessary to capture morphological differences indicative of the cells being alive or dead, and more subtle differences indicative of senescence, for example. To prove the capability of capturing morphological structures, fluorescent human fibroblasts were imaged using a 100× objective. Figure 6 shows fibroblasts transfected with eGFP that had been grown for four days on a standard coverslip prior to imaging. In Figure 6 the nucleolus and other details inside the nucleus can be observed. The fibrous cytoskeleton of the cell was also appreciated, proving that the imaging system was able to accurately capture details at the subcellular level.

## 4. Discussion

We developed an imaging system capable of accurately detecting fluorescent particles inside PDMS microfluidic devices. In the literature, only a few works explain in detail the development of a complete system for fluorescence microscopy, as explained, for instance in Kim et al. [30]. As shown in Figure 4, the imaging system is able to image particles smaller than most bacteria, both using 100× and 20× magnification objectives. This is significant as it means that bacteria in the range of a few micrometers will be accurately imaged using the proposed set up. Having two different objectives allows us to obtain information both at the population level as well as at single-cell analysis. Using the 20× objective population information such as total fluorescence related to the number of living cells can be obtained, while the 100× objective can capture details at the subcellular level related to the single-cell viability.

Regarding the fluorescence detection system, the camera that is currently installed is the Allied Vision Manta G-145B NIR CCD Camera. It is important to notice that the currently mounted camera fulfills the desired purpose, but it also presents several capabilities, such as the near infrared optimization that increase its cost and are not required for the described application. It was used as the sensor for this imaging configuration as a proof-of-concept. In a similar vein, the achromatic doublet used as a condenser lens could be substituted for a less expensive aspheric condenser lens. As the final objective of the microscope is to optimize the cost without compromising the specifications required, other sensors that fulfill this function at a lower cost should be studied in a further development of the system. 

Our motivation to build this microscope is to image bacteria inside microfluidic devices. Bacteria sizes vary widely among different species, but the general consensus is that spherical bacteria range between 0.5–2.0 µm. In the case of bacteria with rod shapes, such as *Mycobacterium tuberculosis*, the specific pathogen for which this imaging system was designed, the length normally ranges between 1–10 µm and the diameter between 0.25–1.0 µm [31]. *Mycoplasma gallicepticum*, at 200–300 nm is the smallest known bacteria [32]. Imaging fluorescent microspheres at the relevant range of sizes ensures that bacteria of all strains could be imaged using the proposed system, providing a powerful tool for the study in a wide range of bacterial diseases. We acknowledge the limitations of this study, as no experiments were conducted with bacteria. Images obtained of fluorescent microspheres were considered as a reliable model to demonstrate the detection capabilities of the proof-of-concept imaging system. The ability to detect particles in the 100 nm to 1 μm range proves that the optical setup would enable antibiotic susceptibility studies with bacteria. However, several requisites need to be addressed before the developed prototype can be used in BSL-3 laboratories, including an environmental chamber surrounding the sample to keep a constant temperature and CO2 concentration, essential for bacterial survival.

This imaging system is specifically designed to detect bacteria, but it has been proven to be suitable for imaging other type of living microorganisms. In the case of eukaryotic cells, we have shown that we can image specific subcellular structures such as the fibrillar structure inside cultured fibroblast (Figure 6). Although further testing using microdevices for eukaryotic cells should be done, the capabilities demonstrated open a wide variety of possible uses. As an example, with fluorescent tagging of specific cell structures, single-cell morphology analysis could be performed in real-time in response to a stimulus. As the cell biology field moves from culture analysis to single-cell analysis, microfluidic devices coupled with custom made fluorescence could prove to be a valuable tool. 

Similarly, the microscope has been demonstrated to be a robust and cheap tool for imaging microorganisms inside microfluidic channels, which opens a broad range of applications related with organ-on-a-chip technologies.

### Future Work

The developed imaging system was built as a proof-of-concept for the stated aims; however, several modifications and improvements are necessary for the system to be implemented in BSL3 facilities where the antibiotic testing experiments take place. The most relevant one is, as mentioned before, the integration of an optimal sensor that fulfills the specific requirements of the antibiotic susceptibility experiments to be performed and the bacteria to be used in those. The future sensor to be incorporated into the system will require a high sensitivity to capture low-light levels of fluorescence coming from the bacteria and, preferably have a moderate cost to be in line with the objective of developing a high-resolution imaging system with a lower cost and smaller size than commercial fluorescence microscopes. Scientific complementary metal-oxide-semiconductor (sCMOS) digital sensors are a solid option as they present high signal-to-noise ratio, high quantum efficiency, high resolution and a moderate cost. As a result, researchers performing long-term monitoring and single-cell analysis of living cells increasingly prefer sCMOS sensors over other digital sensors like CCDS or CMOS.

An additional aspect to be considered is the development of a point-visiting application that, together with an autofocus capability, would allow the automatic recording of the microchambers integrated in the microfluidic devices during the time-lapse experiments. This combination of capabilities is needed in order to use the imaging system on multiplexed microdevices, as time lapse images need to be acquired from the several experiments being carried out concurrently in the same microdevice. 

Finally, for the analysis of bacterial colonies, especially for high pathogenic organisms as the object of this study, *Mycobacterium tuberculosis*, the microscope would be operating inside an environmental chamber or a biosecurity cabin (BSL-3 or BSL-4). The space in this type of laboratory is highly limited, so the small volume of the microscope poses a great advantage, ensuring that several of them could be placed in a reduced amount of space. 

## 5. Conclusions

In this work, we have developed an epifluorescence inverted microscope for imaging bacteria inside microfluidic devices during antibiotic susceptibility testing. For antibiotic drug development, multiplexing is pursued to increase the efficiency of the technique, but the level of multiplexing within the microdevice is limited by the area of acquisition of the microscope. 

Traditionally, multiplexing at the microscope level is not desired due to the elevated cost of these devices. Developing a custom-made special-purpose microscope ensures that the final set up only presents the combination of features required for our purpose and if self-assembled, it provides a cost reduction compared to commercially available microscopes. Through the combination of the suitable image sensors, lens, objective, and filters we have demonstrated the viability of our device for the desired purpose. In this part of the design, the sensor used for the image detection is a commercial CCD camera. Particles around 100 nm have been accurately detected with the microscope, which is small enough for the purpose of this work.

To study bacterial colony dynamics is necessary to capture time-lapse microscopy images. We have demonstrated that our custom-made microscope is capable of imaging 1 μm fluorescent microspheres in suspension inside the chambers of a microfluidic device. The resolutions of the imaging system enabled the accurate detection of the microspheres, what leads to the assumption that bacteria could be easily detected, tracked and their proliferation analyzed with the developed microscope.

We hope that this work to be useful for other research groups studying bacteria inside microfluidic devices and wanting to manufacture a simple, easy to assemble microscope that is cost-effective but also has the necessary capabilities required for their specific research applications.

## Figures and Tables

**Table 1:** Imaging components depicted in Figure 1.

Number	Component	Model
1	Blue LED	LUMILEDS blue light-emitting diode.
2	Glass Ground Diffuser	Thorlabs Unmounted N-BK7 Ground Glass Diffuser DG10-220
3	Condensing Lens: achromatic doublets lens	Thorlabs AC254-030-A
4	Excitation Filter	Thorlabs MF469-35
5	Tunable lens	Optotune EL-16-40-TC
6	Magnification Objective	Either Olympus UPlanFLN 100× or Motic CCIS Plan achromatic phase objective UC Ph2 20×
7	Dichroic Mirror	Thorlabs MD498
8	Dielectric Turning Mirror	Thorlabs CCM1-E02/M 30
9	Emission Filter	Thorlabs MF525-35
10	Cage Adapter	Thorlabs LCP02/M
11	Tube Lens	Thorlabs TTL180-A
12	Cage Adapter	Thorlabs LCP01/M
13	C-mount Adapter	Thorlabs SM2A31
14	CCD Sensor	Allied Vision Manta G-145B NIR CCD Camera
15	Post holder	
